# What Interrupts Suicide Attempts in Men: A Qualitative Study

**DOI:** 10.1371/journal.pone.0128180

**Published:** 2015-06-19

**Authors:** Michael J. Player, Judy Proudfoot, Andrea Fogarty, Erin Whittle, Michael Spurrier, Fiona Shand, Helen Christensen, Dusan Hadzi-Pavlovic, Kay Wilhelm

**Affiliations:** 1 Black Dog Institute, Sydney, Australia; 2 Centre for Research Excellence in Suicide Prevention, Sydney, Australia; 3 Faculty of Medicine, University of New South Wales, Sydney, Australia; 4 Faces in the Street, O’Brien Centre, St Vincent’s Hospital, Darlinghurst, Sydney, Australia; Medical University of Vienna, AUSTRIA

## Abstract

Despite higher rates of suicide in men, there is a dearth of research examining the perspectives and experiences of males at risk of suicide, particularly in terms of understanding how interventions can be tailored to men’s specific needs. The current study aimed to examine factors assisting, complicating or inhibiting interventions for men at risk, as well as outlining the roles of family, friends and others in male suicide prevention. Thirty-five male suicide survivors completed one-to-one interviews, and forty-seven family and friends of male suicide survivors participated in eight focus groups. Thematic analysis revealed five major themes: (1) development of suicidal behaviours tends to follow a common path associated with specific types of risk factors (disrupted mood, unhelpful stoic beliefs and values, avoidant coping strategies, stressors), (2) men at risk of suicide tend to systematically misinterpret changes in their behaviour and thinking, (3) understanding mood and behavioural changes in men enables identification of opportunities to interrupt suicide progression, (4) distraction, provision of practical and emotional supports, along with professional intervention may effectively interrupt acute risk of harm, and (5) suicidal ideation may be reduced through provision of practical help to manage crises, and helping men to focus on obligations and their role within families. Findings suggest that interventions for men at risk of suicidal behaviours need to be tailored to specific risk indicators, developmental factors, care needs and individuals’ preferences. To our knowledge this is the first qualitative study to explore the experiences of both suicidal men and their family/friends after a suicide attempt, with the view to improve understanding of the processes which are effective in interrupting suicide and better inform interventions for men at risk.

## Introduction

While many more women attempt suicide, suicide rates are significantly higher in men [1,2]. Current research suggests that the way men express and cope with their depression or distress, and whether they choose to seek help for their mood is also different to that of women [3,4].

Rather than expressing or identifying with feelings such as stress, sadness and emptiness, men typically demonstrate numbing (e.g. alcohol and drugs), risky (e.g. gambling, violence), defensive (e.g., anger, aggression) or otherwise avoidant (e.g., social withdrawal) types of coping behaviours [3] relative to women. Furthermore, a positive correlation is observed between higher adherence to masculine norms and a higher likelihood of experiencing depressive episodes [5,6].

In general, men are much less likely to seek help for depression and other mental health issues [7,8,9]. A similar pattern has been reported with suicide. In the 12 months prior to a suicide, 35% of men compared to 58% of women sought care from a mental health practitioner [10]. Additionally, men are at higher risk of dying by suicide owing to ‘acquired capability’, evidenced by habituation to fear and pain, and use of more lethal methods of self-harm than women [11,12,13].

To date, there has been a lack of research that focuses on understanding the perspectives and experiences of men who have attempted suicide [14,15]. There has also been little research investigating whether suicidal men require substantively different approaches to treatment and intervention [16].

In an attempt to reduce the burden of male suicide, the present study provides a qualitative investigation of male suicidality—which includes ideation and behaviours, focusing on factors which interrupt a suicide attempt. Specifically, the project aims to: (1) describe contributing, protective and preventive factors that assist, complicate, restrict or inhibit interventions for men at risk of suicide, and (2) outline the role of family, friends and others in prevention of suicide in men, to improve interventions specifically targeting suicidal men.

## Methods

### Participant recruitment

Recruitment was conducted in each state and territory in Australia, and targeted two specific groups using a purposive sampling methodology: (i) men who had survived a suicide attempt between 6 and 18 months prior to recruitment, and (ii) men and women who were family or friends of men who had survived a suicide attempt 6 to 18 months prior to recruitment. The time frame of 6–18 months was selected to minimize risk of triggering significant distress around unresolved feelings, while enabling recall of subtle and significant details of the situation leading up to suicide attempt.

The study was publicised through national and state-based mental health organisations, communication and professional networks, as well through community-based organisations in each state and territory.

Individuals who expressed interest in participating were initially screened via telephone to assess their current mental health and suitability to take part in the study. Those who met inclusion criteria provided written informed consent. All participants were reimbursed $50 for their time and travel expenses.

### Data Collection

Before commencing the interview or focus groups, participants completed a brief questionnaire containing basic demographic information and three validated scales to assess current risk of, and levels of depression and anxiety. The Patient Health Questionnaire-9 (PHQ-9) is a nine item self-administered scale used to assess depression severity, by asking how often in the past two weeks participants have been bothered by a range of symptoms or problems [17]. The Male Depression Risk Scale (MDRS) is a self-administered scale of 22 items which assesses multiple domains of ‘externalising symptoms’ associated with male experiences of depression, for example, aggression [18]. The Generalized Anxiety Disorder 7-item (GAD-7) scale is a seven item self-administered scale used to assess the severity of generalised anxiety by asking how often participants have experienced symptoms in the previous two weeks [19]. Participants were also asked about their history of depression, help seeking and use of anti-depressant medication, as well as their current mood, the latter on a visual analogue scale (VAS), which they repeated at the end of the interview/focus group.

An interview schedule was developed from a thorough literature review. It was piloted and adjusted based on feedback from participants. The final schedule explored factors that contribute to making a suicide attempt, those that interrupt an attempt, and the factors that men, family and friends find useful in preventing a future attempt.

The same research officer (MP), conducted all thirty-five interviews and eight focus groups. None of the participants was known to the research officer prior to participation. With participant consent, all interviews and focus groups were digitally recorded.

### Risk Management

Due to the sensitive nature of the research, a robust risk management procedure was developed for the project. It covered screening, identification and management of ‘at-risk’ participants.

### Analysis

Digital recordings were de-identified before being transcribed by an external transcription service. However, to ensure full immersion in the data, two coders (MP, MS) reviewed the transcripts while listening to recordings to check for accuracy and edit for anonymity. In line with thematic analysis guidelines by Braun and Clarke (2006) [20], coding was conducted by two primary coders (MP, MS) using NVivo 10 software (QSR International P/L, 2012). Both coders have clinical psychology training and previous experience conducting interviews and focus groups regarding mental health issues. The data set was coded in a data driven manner, with coding not guided by a preconceived thematic framework. Initial codes were developed individually by the two coders, with the data then broken down into basic elements. To establish commonalities and reliability of coding styles and structure, three transcripts were analysed by both authors. Once a coding framework was agreed upon, the two coders independently coded the remaining transcripts. Data points were collated into categories that formed the initial themes, which were used to identify conceptual relationships between the initial themes. ‘Overarching themes’ which provided a more intricate understanding of the subject matter were then proposed and tested against the data set.

The over-arching themes were discussed with the research team on several occasions to establish an acceptable thematic map of the data [19]. The validity and usefulness of themes and subthemes were regularly evaluated by the coders to ensure consistency and coherency. Final themes and their subthemes were established through a process of consensus within the research team in order to provide a detailed explanation of pathways towards and away from suicidality. Data saturation was reached when all focus group transcripts and three interview transcripts from each state had been coded, with no new codes emerging. The remaining transcripts were not coded line by line but read for consistency with the results.

The study including the consent procedure was approved by the Human Research Ethics Committee of the University of New South Wales Australia (HREC 13077).

## Results

### Sample

Thirty-five men who had made a suicide attempt participated in interviews which lasted between 45–70 mins. There was a median age of 43 years (range 18–67 years). Just over one-third of the men (34%) reported current employment, 46% were unable to work and 20% were unemployed, studying or retired. Just over half (54%) had never been married, 11% were currently married and 35% were separated or divorced.

Forty-seven family and friends of men who had made a suicide attempt participated in focus groups lasting between 60–90mins. Twenty-six (55%) were female, with a median age of 47 years (range 19–65 years). In contrast to the male interviewees, a higher proportion reported current employment (53%), with only 19% unable to work and 27% being unemployed, studying or retired. Nearly half (49%) of family and friends were currently married or in a de-facto relationship, 28% were never married and 23% were separated, divorced or widowed.

Both family/friend participants and individual male participants had mean scores within the mild range of depression on the PHQ (*M* = 5.5, *S*.*D*. = 5.7 and *M* = 8.0, *S*.*D*. = 6.2 respectively), with family/friend participants in the none to minimal, and male participants in the mild range of anxiety as scored on the GAD (*M* = 4.1, *S*.*D*. = 4.6 and *M* = 6.3, *S*.*D*. = 5.6 respectively). A majority of the family/friends (60%) and the males (83%) reported they had felt down, lost pleasure in things and experienced difficulty cheering up in the last two weeks, with 51% and 80% having seen a health professional for these health problems respectively. Fifty-five percent of family/friends and 94% of men had taken medication for depression. A majority of the family/friends (87%) and males (71%) reported they had not experienced any suicidal thinking in the two weeks prior to participation.

No participants dropped out from the study nor requested their data to be removed from analysis.

### Thematic analysis

The same thematic structure was observed in the data from both the interviews and focus groups, therefore it was possible to combine the data. Results of the thematic analysis identified five major themes, namely, (i) Pathways to a suicide attempt, (ii) Men at risk, (iii) Successful intervention through greater understanding, (iv) Interrupting strategies for acute and immediate risk (v) Protective factors and preventative strategies. Four of the major themes had sub-themes, as outlined below.

#### (i) Pathways to a suicide attempt

Although there were occasional references to an innate pull towards suicide, most participants reported an idiosyncratic path to suicide beset with adversity, distress and poor decision-making.

#### Risk factors for male suicide

Nearly all male participants reported experiencing some or all of the following four factors in the development of suicidality:

- a period of depressed or disrupted mood
- unhelpful conceptions of masculinity consisting of stoic beliefs and values, which strongly influenced decision-making
- social isolation and use of other avoidant coping strategies
- at least one, but often many, personally meaningful stressors.

These elements tended to interact with each other and worsen over time, leading to greater suicide risk, whilst also creating barriers that interfered with attempts to treat depression or interrupt suicidality.

A depressed or disrupted mood was described by most participants to coincide with some or all of the following symptoms: decreased motivation and activity, poor concentration, tiredness, irritability and agitation, changes in sleep and appetite, and perceiving life incidents negatively. Disrupted mood often included an increase in anger, aggression and violence, social isolation, as well as increased suicidal thinking. Participants reported that acute suicidal thinking was associated with excessive risk-taking, overt statements of intent, hopelessness, apathy or appearing ‘at peace’.

A common observation was that beliefs and values deriving from adherence to the ‘stereotypical’ stoic Australian male identity were unhelpful, especially when held in extreme forms. Almost all men reported that their masculine beliefs led to them isolating themselves when they were feeling down, for example, to avoid imposing on others. Failure to manage emotions, or live up to expectations of happiness or coping also often led to a sense of lost control or guilt, as well as anxiety about having these perceived weaknesses or failures revealed. It was very common for family and friends to state that this tendency of men to adopt typically masculine responses to distress meant that they were often unaware of warning signs for suicide, or misinterpreted suicidality as depression or anger.

Both groups agreed that stoic belief systems, deficits in knowledge or emotion regulation skills, combined with the depletion of cognitive resources associated with depression, typically led men to select unhelpful coping strategies. Many men stated that their attempts to manage problems to avoid revealing weakness or stigmatising labels, led them to isolate themselves and instead rely on coping strategies that required less immediate effort and provided short-term alleviation of problems, for example, drug or alcohol use, gambling, and working excessively. However, these strategies repeatedly made problems worse in the long term through, for example, debt creation, and emotional reaction and interpersonal conflicts.

Several friends and family members also observed that depressed and suicidal males often expressed anger or aggression instead of, or to avoid feelings of sadness, anxiety or stress. However, angry, aggressive or defensive behaviour was also likely to strengthen social isolation by increasing risk of interpersonal conflict, reinforcing a sense of disconnection, and lowering motivation for family and friends to offer help.

Male participants also reported that when they were vulnerable to suicidal thoughts due to disrupted mood, stoic views or avoidant coping choices, personally meaningful stressors tended not to resolve, took on greater significance, or compounded in unhelpful ways. A majority of participants noted that ordinarily manageable stressors then tended to cumulatively degrade mood and functioning, sometimes into a state of acute, actively suicidal behaviour.

#### Downward spiral

There was broad agreement among men, family and friends on the process by which men became suicidal, with many participants noting that any stressor, meaningful or not, had the potential to tip a man over the threshold from disrupted mood to suicidality depending on the chronicity of his mood state. Men often reported that the actual stressor that triggered a suicide attempt was not necessarily of great personal significance, with some family and friends supporting this view. As one focus group participant described: ***“*** *Quite often it seems like it was more things that compound*, *and that it would just be one little thing that would trigger it off that normally would be okay but because of that compounded effect*, *it made it not okay”*. *(Focus group participant*, *Male*, *19)*. Thus a suicide attempt was often the result of a series of stressors over an extended period of time. Men repeatedly noted how they felt unable to change the momentum of negative life events, and described feelings of hopelessness and despair that this situation would never change.

Additionally, some reported that the adherence to masculine norms meant that sometimes the feelings associated with being vulnerable were more anxiety provoking than the thought of being dead. Ambivalence therefore featured prominently in the ‘downward spiral’, with men often opting for suicide rather than asking for help.

#### Time to die

Many men described a similar pattern to their suicide attempts. The ‘downward spiral’ in mood and activity resulted in them becoming vulnerable to hopelessness and suicidal thoughts, with a period of planning and preparation preceding the actual decision or commitment to attempt suicide. Finally, the men hit bottom, crossing a threshold of distress or willingness to persevere and they became acutely suicidal, at which time they made attempts to suicide.

*“It’s like you’re wrestling with it*, *and it’s not just for a day…*.*it can build up like for months*, *and it’s to the point where it torments you*, *where there is almost at times no rest*, *and this is constantly been on my mind*. *So–and up until that period I suppose I’ve been looking for some kind of answer*. *So once I get to there and I’ve decided*, *I’ve pretty well made up my mind that there’s no help”*. *(Interview participant*, *Male*, *40)*.

#### (ii) Men at Risk

At risk individuals, and those close to them, reported that they tended to systematically minimise disturbed mood or changes in behaviour, either avoiding, or failing to identify associated risk and habituation to suicidal thinking. As a result, interventions may have come only after significant risk of harm and/or roadblocks to intervention had been able to develop.

**What is this I’m experiencing?** The majority of interviewed men described how they routinely denied or misinterpreted their disrupted mood. At the time, they didn’t make a link between their externalising behaviours, their mood and an increasing risk of suicidality. This was supported by focus group members who noted it was difficult for men to identify when they were at risk, for example:

*“a psychiatrist described to him (that) what he was going through was trying to hunt for pink diamonds*. *He may not understand what it is but he knows they’re in there somewhere and he’s got to keep hunting for them because understanding that or identifying them will help him understand that he’s in a moment of being depressed or needing help*, *and to acknowledge it”*. *(Focus group participant*, *Female*, *48)*.

#### Well-worn thought pattern

Many men described that while in a vulnerable state their thoughts would habitually turn to suicide, and the constancy of these thought patterns normalised the idea of suicide in their minds, such that they began to perceive it as a viable option. This pathway may thus have become a well-worn groove in their thinking.

“….it *(suicide)* will ebb and flow too….So, on the days I was really down, I would be thinking or feeling very helpless and hopeless and thinking, well, that could be a way out. And then the next day I would be fine. But I’d talk myself into being fine”. (Interview participant, Male, 53).

#### Slipping through the cracks

Family and friends frequently reported not recognising depressed moods in men, as these moods often did not conform to expectations of what depression should look like. Instead, men expressed their low mood via externalising behaviours which complicated family and friends’ understanding of men’s risk, such that they were unsure how to respond. Participants noted that when it was clear that a man was suicidal, he was often highly agitated and distressed. Many family and friends at that point felt overwhelmed and this limited their responses for fear of saying the wrong thing or making the situation worse.

“I would just isolate myself….it got to the point where they didn’t know what to do. I became so moody and unpredictable that nobody wanted to intervene because they didn’t know what direction that would send me. They’d have to sit on the outside and say, “We love you mate, but we’re here for you, but we’re not going to push you to do anything.” They were very worried that if they tried to become controlling or demanding that it would tip me over the edge”. (Interview participant, Male, 26).

Once there was awareness of a man’s suicidality, family and friends described how they felt real risks and consequences for choosing to act, or choosing not to act, which in some cases resulted in men ‘slipping through the cracks’.

*“We didn’t probably take it serious enough when he was asking for help*. *And it’s a horrible thing to say*, *but we all work and we’re all busy and you’ve all got your own life and at the time I was just*, *you know*, *I’m busy*, *leave me alone*. *I shouldn’t say that but I did*. *I felt guilt for a very long time after that I didn’t give him more attention”*. *(Focus group participant*, *Female*, *53)*.

#### (iii) Successful intervention through greater understanding

A major theme emerging from the data was that deeper understanding of men’s specific moods, symptoms and progression into suicidality may illuminate opportunities to interrupt the pathway to suicide. Nearly all focus group members reported that effective monitoring of and appropriate responding to men’s warning signs was crucial to keeping men safe. This becomes even more important in light of disclosures from most men who often did not acknowledge distress or low mood, and only rarely admitted their intent to attempt suicide.

Successful interruptions were discussed by men and family/friends as targeting the four core features of suicidality (disrupted mood, unhelpful stoic beliefs and values, avoidant coping strategies, and stressors). Effective interventions were also described as those that either aimed to improve men’s internal coping and resilience through improvements in self-care, emotion regulation and communication skills or involved external care systems such family and friends, community and mental health professionals. However, the best results were achieved when the intervention strategy was paired to the level of risk articulated by their warning signs as described below.

#### (iv) Interrupting Strategies for Acute and Immediate Risk

Interrupting strategies were those that kept men safe from the immediate danger of suicide when at maximum risk. Family and friends reported success in supporting men through these acute moments using strategies mainly based upon providing distraction, and practical and emotional support, sometimes ‘around the clock’. If these attempts failed, or if the man had become too risky for these strategies, then mental health professionals were contacted so men were unable to harm themselves.

#### Distraction

Family and friends reported having modest goals during periods of acute suicidality. Keeping a man distracted, even for an hour or two, was crucial in providing space so he wasn’t actively planning his attempt. Men noted that this relieved pressure and acknowledged that while the distraction was normally not enough to fix the problem, it provided crucial respite from their suicidal thoughts. For young men distraction might involve participating in a high adrenaline activity such as go-karting, to *“totally blast everything out of their brain other than that moment of joy” (Focus group participant*, *Female*, *48)*.

Other men described that being distracted or motivated by a responsibility to others would interrupt their suicidality.

*So a couple of days later a mate of mine needed a lift to the airport*. *And I was like*, *I’ve got to do that*, *he’s my mate*. *And for two days I didn’t even think about it (suicide)*. *And that was rare*. *So having some sort of connection*, *having some sort of interaction…*.*almost an obligation to something”*. *(Interview participant*, *Male*, *28)*.

However most men at some point described losing their will to act for their own benefit, and at these times it was essential that others intervene.

#### Seeking professional help

While a large number of participants from both groups reported unsatisfactory experiences with community or health services, and mental health professionals directly, there was agreement that they often played a vital role in helping individuals at various stages and importantly, at critical periods of risk. Family and friends considered these services to have a greater knowledge, skills and capacity to manage and contain men ‘at risk’, while simultaneously reducing the immediate pressure and responsibility they felt.

#### (v) Protective Factors and Preventative Strategies

Participants revealed a separate class of intervention strategies that were thought to keep men safe from acting on recurrent suicidal thoughts, which had not yet reached a critical stage. Predominantly the protective factors described by men were thoughts or reminders of the anticipated impact on their family of the suicide, but also the receipt of practical help to manage life crises.

#### Family and Legacy

It was common for men to report feelings of guilt and selfishness with regard to the effect their suicide would have on family and friends. For example:

*“…*.*what I felt about hurting my old man by doing it I did feel across the board for everyone that I knew*, *they’d all be hurt*. *But often I would visualise my own funeral*, *and I visualise how many people would actually come to it*, *am I really that worthless*? *And then I’d sort of go through in my head how many people you’re actually going to hurt by doing this*. *And*, *yeah*, *there was a fair few people sitting at those imaginary funerals crying and not understanding why that I’ve gone and done this when I didn’t have to*. *And a lot of them I visualise in my head saying*, *why didn’t he come to me*? *I had no idea he had this problem*. *So that was a good way of stopping it from happening”*. *(Interview participant*, *Male*, *26)*.

In discussing the ‘legacy’ they would leave behind in the event of their suicide, men often thought about their children and any questions they might have. For one man, this included worrying that, as a direct result of their own suicide, his children may experience a sense of worthlessness which could in turn elevate the risk of their children considering suicide at some point in the future:

*“…leaves a lot of questions for a child left behind by a suiciding parent*. *There’s a lot of doubt*, *did they love me and they didn’t love me enough to hang around*? *And the idea that the child when they’re down*, *they think that that’s an option as well…”(Focus group participant*, *Male*, *39)*.

While not all men described experiencing these thoughts, children nevertheless were described as a ‘protective’ factor that could be effective in helping men ‘pause’ on a pathway to suicide.

#### Providing practical help to men

A number of focus group participants gave examples of men who had responded to interventions which focused on the stressors that triggered the suicidal thoughts. They highlighted that helping men plan how to confront tasks that they may have neglected, might be a means by which they regain some control and agency, and re-engage them in living.

*“Because the psychological point of view*, *it has its uses*, *but blokes are physical and practical*, *and they want a way out of the situation that’s caused them to get there*, *not just*, *oh*, *there*, *there*, *mate*, *you’ll be right”*. *(Interview participant*, *Male*, *67)*.

Many family and friends advised that it was important to find ways to help build a man’s ‘positive momentum’. They felt it was important to set small, achievable goals so that a man experienced regular small wins, which created forward momentum.

*“…*.*and after I had the initial*, *no*, *no*, *no*, *no*, *no; which is the whole world can’t be fixed; when I pushed him and some leading questions he started to come up with some solutions…*. *And as soon as he realised he’d come up with the idea and it could happen*, *he tackled it in a different way and he’s come out…with a smile on his face*. *But he solved it—I knew what was possible*, *but I gave him some leading questions to let him then figure it out for himself*, *and that seems to have worked”*. *(Focus group participant*, *Female*, *48)*.

#### Respected people

A separate class of preventative strategies that halted or reversed the downward spiral were described by both participant groups. Men reported benefiting from regular contact with a person with whom they could discuss problems, providing space to ventilate and address distress. They identified that respect for, or having trust in these support persons was crucial, and that they could be more beneficial if they are outside the immediate family. There were often cultural considerations involved in who was deemed to be an appropriate support person.

“I want to know that people still placed some value on my friendship or my interaction in their life or whatever. So I want to hear that, probably, from close friends. So where I think that one step away yet still very close in the point of close friends to say—come and say hey, we love you. We love who you are. I guess, that beats for me a professional saying something and I think it beats for me a family (member) saying it because a family is going to say it all the time”. (Interview participant, Male, 37).

“Culturally, I did a lot of work with Aboriginal families in the Northern Territory, with tradition, and that was the biggest issue. They would only accept certain people being involved, or even knowing about any of this sort of stuff”. (Focus group participant, Female, 57).

#### Emotional regulation and coping

Both participant groups consistently described consulting a mental health professional as an important part of the process for maintaining men’s moods. However, results indicated that successful changes to emotion regulation and coping were often an idiosyncratic process. Broadly, while shame and embarrassment ensured that some men only felt comfortable talking about mental health issues with confidential support services such as *Lifeline*, *Beyondblue* and *Alcoholics Anonymous*, other men needed to have the support and understanding of family and friends.

## Discussion

The current study revealed patterns of suicidal behaviour associated with specific risk factors and warning signs in men, combining within a pathway to suicide consisting of a number of common features. These contributors have been categorised within the literature in relation to a number of core risk factors: mental illness, low individual resilience, and recent stress—that combine to trigger suicidal behaviours [21,22]. The current study confirmed that men at risk of suicide similarly demonstrate vulnerability to these common types of risk factors (mental health problems, low resilience, stress), but did so in a manner framed by specific masculine neurobiological and sociocultural context [23].

The way men experienced stressful events, for example, appeared to relate to aspects of masculine identity (e.g., stoic beliefs associated with avoidant, isolative coping strategies), and psychological context (e.g., affective or substance abuse issues more prevalent among men[24]. Males therefore tended to experience mental illnesses and process stress in a manner making them likely to engage in violent or physically risky behaviours, isolate, use alcohol and drugs, or otherwise fail to address problems until suicidal ideation had reached late stages of development. At this late stage men who may be prone impulsivity and reduced fear of pain and death, referred to as ‘acquired capability’, may be at risk of acting on ideation [13]. Therefore, cognition and behaviour associated with male suicide took on forms idiosyncratic to gender, supporting observed differences in vulnerability to risk and harm (e.g., higher levels of self-harm in females, lower disclosure of suicidal ideation and higher rates of death by suicide in males) [23,25,26].

The effectiveness of intervention for this subgroup was therefore determined by how well approaches addressed key features contributing to suicidal behaviour [27], or decision making changes occurring as men approached more acute stages of suicidality [16,28]. Effective intervention strategies unsurprisingly often overlapped with evidence-based treatment guidelines relevant to psychological disorders identified by participants as contributing to suicidal behaviours (e.g., mood, anger, stress, or personality problems) [29,30,31].

Findings from the current study have a number of implications for clinical policy and practice. Specifically, resources and approaches need to address core cognitive-behavioural features of suicidal males such as coping and emotion regulation skills, match idiosyncratic presentations of warning signs, and take into account the unique roles played by the different stake-holders, including family/friends in this process. The current study suggested differing roles for key players in suicide prevention and intervention. For example, it is worthwhile for family and friends to specifically address diverting men at risk away from risk situations and alcohol and substance use when they are down.

Individuals may arrest, avoid, or reverse either the downward spiral in mood and functioning, or acute suicidal behaviour through various behavioural strategies used to improve mood, manage relationships and distress, or solve problems. Effective behavioural strategies increase activity, social connectedness, or both. For example, moods and functioning were typically improved via behavioural strategies that broke up routine and boredom, increased social contact, rewarded self-esteem (e.g., performing altruistic acts) or returned individuals to relationships or activities that they had previously enjoyed, or which had positive physical, physiological or neurophysiological impacts (e.g., exercise, healthy diet, reduction in drug and alcohol use).

Individuals’ immediate social network may also play an important role in protecting men, or intervening as men became suicidal. Increasing individuals’ contact with others appeared important to recovery for the majority of suicidal males and is consistent with findings that social connectedness is a protective factor. Contact allowed men to learn new communication and coping skills, ventilate and normalize distress, and disclose important risk information, as well as allowing men to feel connected, and acknowledge obligation to and care from others. A number of individuals noted that realisation or acknowledgment of obligation or connection to family or close friends represented beliefs inconsistent with death, critical in protecting men during periods of acute suicidality, and therefore valuable as ideas to promote among men at risk.

Mental health professionals in community health or emergency departments must be encouraged to perform suicide risk assessment in men who present with externalising behaviours and adjustment features, asking questions to further identify these typical male risk-taking behaviours [32]. Community services may assist in male suicide intervention and prevention via: psychological or medical treatment for disorder, psychoeducation, and connection to supports that change males’ beliefs and behaviours, or help men to cope with stressful events. This may include using patient ambivalence about dying, noted to be a key area for intervention ([33]. Participants reported that it was not only traditional health and allied government services that assisted men in this manner, but often community groups or individuals not specifically designated to such roles (e.g., fellow members of mental health support groups).

Several participants noted that limiting individuals’ capacity to choose death was sometimes essential for protecting men against self-harm, and providing opportunity for intervention and change. This might be managed through involuntary admission to a mental health inpatient unit, for example. A number of individuals and focus group members acknowledged however that responsibility taken for males’ safety by family, friends or external services could only ever temporarily arrest suicidal behaviour, and that males needed ultimately to take responsibility, choose life, and change habitual cognitions and behaviours in order to experience real, long term recovery from suicidality.

Despite researchers’ adherence to a scientific methodology [34], it is difficult to apply insights from this study to other suicidal men, due to fact that was a qualitative study which, by definition, involves an in-depth exploration within a small non-representative sample and inevitable bias in participant and researcher viewpoints. We found that this was a very unique and difficult population to sample, due to the specific time parameter for the man’s suicide attempt, designed to ensure accurate recall yet also enabling reflections not confounded by trauma or grief.

Explanations throughout the study related to suicidal behaviours, without control for combinatory effects among all relevant variables. Without psychometrically validated, quantitative measurement, it is difficult to reasonably infer which variables influence each other, or decisions, particularly when variables reportedly played different roles for different individuals. Future research is therefore clearly needed to validate and build upon the preliminary findings outlined here. Specifically, larger samples and mixed qualitative–quantitative data collection methods should be employed to further expand and test the findings of this study.

## Conclusion

The current study offers some preliminary data suggesting that development of suicidal behaviours in males is associated with a number of core risk factors, developing via a specific pathway characterised by sociocultural and biological gender differences. Theoretical models and corrective interventions for suicidal males need to be adapted, and suicidal behaviour may be reduced, by addressing common risk factors, in conjunction with supporting changes to men’s decision-making that occurred as men approached acute states of suicidality.
